# *GSTP1* and *TNF* Gene Variants and Associations between Air Pollution and Incident Childhood Asthma: The Traffic, Asthma and Genetics (TAG) Study

**DOI:** 10.1289/ehp.1307459

**Published:** 2014-01-24

**Authors:** Elaina A. MacIntyre, Michael Brauer, Erik Melén, Carl Peter Bauer, Mario Bauer, Dietrich Berdel, Anna Bergström, Bert Brunekreef, Moira Chan-Yeung, Claudia Klümper, Elaine Fuertes, Ulrike Gehring, Anna Gref, Joachim Heinrich, Olf Herbarth, Marjan Kerkhof, Gerard H. Koppelman, Anita L. Kozyrskyj, Göran Pershagen, Dirkje S. Postma, Elisabeth Thiering, Carla M.T. Tiesler, Christopher Carlsten, for the TAG Study Group

**Affiliations:** 1 School of Population and Public Health, University of British Columbia, Vancouver, British Columbia, Canada; 2 Institute of Epidemiology I, Helmholtz Zentrum München, German Research Centre for Environmental Health, Neuherberg, Germany; 3 Institute of Environmental Medicine, Karolinska Institutet, Stockholm, Sweden; 4 Sachs’ Children’s Hospital, Stockholm, Sweden; 5 Department of Pediatrics, Technical University of Munich, Munich, Germany; 6 Department for Environmental Immunology, Helmholtz Centre for Environmental Research–UFZ, Leipzig, Germany; 7 Department of Pediatrics, Marien-Hospital Wesel, Wesel, Germany; 8 Institute for Risk Assessment Sciences, Utrecht University, Utrecht, the Netherlands; 9 Julius Center for Health Sciences and Primary Care, University Medical Center Utrecht, Utrecht, the Netherlands; 10 Department of Medicine, University of British Columbia, Vancouver, British Columbia, Canada; 11 Leibniz Research Institute for Environmental Medicine, University of Düsseldorf, Düsseldorf, Germany; 12 Environmental Medicine and Hygiene, University of Leipzig, Leipzig, Germany; 13 Department of Epidemiology, University of Groningen, University Medical Center Groningen, Groningen, the Netherlands; 14 Department of Pediatric Pulmonology and Pediatric Allergology, Beatrix Children’s Hospital, University of Groningen, University Medical Center Groningen, Groningen Research Institute for Asthma and COPD (GRIAC), Groningen, the Netherlands; 15 Department of Pediatrics, Faculty of Medicine & Dentistry, University of Alberta, Edmonton, Alberta, Canada; 16 School of Public Health, University of Alberta, Edmonton, Alberta, Canada; 17 University of Groningen, Department of Pulmonology, University Medical Center Groningen, Groningen, the Netherlands; 18 GRIAC, Groningen, the Netherlands; 19 Division of Metabolic Diseases and Nutritional Medicine, Ludwig-Maximilians-University of Munich, Dr. von Hauner Children’s Hospital, Munich, Germany

## Abstract

Background: Genetics may partially explain observed heterogeneity in associations between traffic-related air pollution and incident asthma.

Objective: Our aim was to investigate the impact of gene variants associated with oxidative stress and inflammation on associations between air pollution and incident childhood asthma.

Methods: Traffic-related air pollution, asthma, wheeze, gene variant, and potential confounder data were pooled across six birth cohorts. Parents reported physician-diagnosed asthma and wheeze from birth to 7–8 years of age (confirmed by pediatric allergist in two cohorts). Individual estimates of annual average air pollution [nitrogen dioxide (NO_2_), particulate matter ≤ 2.5 μm (PM_2.5_), PM_2.5_ absorbance, ozone] were assigned to each child’s birth address using land use regression, atmospheric modeling, and ambient monitoring data. Effect modification by variants in *GSTP1* (rs1138272/Ala^114^Val and rs1695/IIe^105^Val) and *TNF* (rs1800629/G-308A) was investigated.

Results: Data on asthma, wheeze, potential confounders, at least one SNP of interest, and NO_2_ were available for 5,115 children. *GSTP1* rs1138272 and *TNF* rs1800629 SNPs were associated with asthma and wheeze, respectively. In relation to air pollution exposure, children with one or more *GSTP1* rs1138272 minor allele were at increased risk of current asthma [odds ratio (OR) = 2.59; 95% CI: 1.43, 4.68 per 10 μg/m^3^ NO_2_] and ever asthma (OR = 1.64; 95% CI: 1.06, 2.53) compared with homozygous major allele carriers (OR = 0.95; 95% CI: 0.68, 1.32 for current and OR = 1.20; 95% CI: 0.98, 1.48 for ever asthma; Bonferroni-corrected interaction *p* = 0.04 and 0.01, respectively). Similarly, for *GSTP1* rs1695, associations between NO_2_ and current and ever asthma had ORs of 1.43 (95% CI: 1.03, 1.98) and 1.36 (95% CI: 1.08, 1.70), respectively, for minor allele carriers compared with ORs of 0.82 (95% CI: 0.52, 1.32) and 1.12 (95% CI: 0.84, 1.49) for homozygous major allele carriers (Bonferroni-corrected interaction *p*-values 0.48 and 0.09). There were no clear differences by *TNF* genotype.

Conclusions: Children carrying *GSTP1* rs1138272 or rs1695 minor alleles may constitute a susceptible population at increased risk of asthma associated with air pollution.

Citation: MacIntyre EA, Brauer M, Melén E, Bauer CP, Bauer M, Berdel D, Bergström A, Brunekreef B, Chan-Yeung M, Klümper C, Fuertes E, Gehring U, Gref A, Heinrich J, Herbarth O, Kerkhof M, Koppelman GH, Kozyrskyj AL, Pershagen G, Postma DS, Thiering E, Tiesler CM, Carlsten C, TAG Study Group. 2014. *GSTP1* and *TNF* gene variants and associations between air pollution and incident childhood asthma: the traffic, asthma and genetics (TAG) Study. Environ Health Perspect 122:418–424; http://dx.doi.org/10.1289/ehp.1307459

## Introduction

Evidence supporting an association between traffic-related air pollution and incident childhood asthma is growing although published estimates highlight variability across different populations. (Anderson et al. 2013; Gehring et al. 2010; Nordling et al. 2008). Genetic variation could be one explanation for this observed heterogeneity and gene–environment studies may help identify those children most susceptible to air pollution (Bråbäck and Forsberg 2009). Large studies that combine multiple birth cohorts are ideal for examining gene–environment interactions in the development of childhood asthma, particularly where there are similar assessments of air pollution and asthma across cohorts (London 2007).

Genes related to oxidative stress are of special interest because of the biological mechanisms thought to underlie both the toxicity of traffic-related air pollution and the development of asthma (Kelly 2003). *GSTP1* (the glutathione *S*-transferase pi gene) codes for an enzyme that metabolizes reactive oxygen species in the lung, and, despite considerable study, associations with atopic disease have not been consistent (Piacentini et al. 2013), possibly because of additional host and environment factors such as age (Salam et al. 2007). To date, *GSTP1* polymorphisms have been reported to modify the association between traffic-related air pollution and allergic sensitization (Melén et al. 2008), persistent wheeze (Schroer et al. 2009), and asthma (Carlsten et al. 2011a) in individual birth cohorts; and in one study these strata-specific findings were further attenuated by *TNF* (tumor necrosis factor) polymorphisms (Melén et al. 2008). *TNF* mediates inflammation and is expressed in response to environmental pollutants (Wu et al. 2007). *TNF* polymorphisms have been associated with asthma (Aoki et al. 2006; Panasevich et al. 2010) and wheeze (Wu et al. 2007) and were previously reported to modify the association between ozone and asthma in healthy young adults (Yang et al. 2008).

We initiated an international collaboration, the Traffic, Asthma and Genetics Study (TAG), to examine the influence of specific candidate genes on the relationship between traffic-related air pollution and the development of childhood asthma.

## Methods

The Canadian Asthma Primary Prevention Study (CAPPS) is a randomized controlled study of high-risk children (Carlsten et al. 2011b). The Study of Asthma, Genes and the Environment (SAGE) is a Canadian population-based birth cohort (Kozyrskyj et al. 2009). The Children, Allergy, Milieu, Stockholm, Epidemiological Survey (BAMSE) is a population-based Swedish birth cohort (Melén et al. 2008). The German Infant Nutrition Intervention Study plus environmental and genetic influences on allergy (GINI) is a population-based birth cohort (Morgenstern et al. 2008). The Influence of Life-Style Factors on the Development of the Immune System and Allergies in East and West Germany plus the influence of traffic emissions and genetics (LISA) study is a population-based birth cohort (Morgenstern et al. 2008). The Prevention and Incidence of Asthma and Mite Allergy (PIAMA) study is a population-based birth cohort (Brunekreef et al. 2002). Detailed cohort descriptions are provided in the Supplemental Material, Appendix 1, and additional details on the TAG study have been previously published (MacIntyre et al. 2013). Parents participating in each cohort provided informed consent, and protocols were approved by all relevant ethical review boards.

Physician-diagnosed asthma and wheeze symptoms were collected from parental questionnaires. CAPPS and SAGE children were also evaluated by a pediatric allergist to confirm asthma. Current asthma and current wheeze were ascertained at 8 years (6 years for GINI/LISA wheeze; 7 years for CAPPS asthma/wheeze). Ever asthma and ever wheeze were defined using every available follow-up to 8 years. Children missing ≥ 2 follow-ups were excluded. Data were collected at slightly different time points, and there were minor differences in question wording across the cohorts (MacIntyre et al. 2013).

Annual average nitrogen dioxide (NO_2_) was estimated using traffic-related land use regression (LUR) models, except for the BAMSE cohort, which used dispersion modeling (Bellander et al. 2001). LUR models developed for PIAMA and LISA/GINI Munich (Germany) were based on data collected in 1999–2000 (Brauer et al. 2003), LISA/GINI Wesel (Germany) in 2003, LISA/GINI Leipzig (Germany) in 2004/2005 (Hochadel et al. 2006), CAPPS Vancouver (BC, Canada) in 2003 (Henderson et al. 2007), and SAGE and CAPPS Winnipeg (MB, Canada) in 2007 (Allen et al. 2010). For all study sites, integrated 14-day sampling campaigns were repeated over a 12-month period to capture seasonal patterns in air pollution. Protocols were specifically developed to spatially model long-term average pollutant concentrations. Potential predictors for traffic were screened based on their correlation with measured pollutants. Final models were assessed based on the coefficient of determination and root mean square error from cross-validation. LUR estimated PM_2.5_ (particulate matter with diameter ≤ 2.5 μm) and PM_2.5_ absorbance (a measure of elemental carbon estimated from filter reflectance measurements) were available for a subset of the combined cohort. For BAMSE, emission data for traffic-generated nitrogen oxides (NO_x_) were collected for the years 1990 and 2000; and dispersion was estimated using a dilution model based on wind speed, direction, and precipitation (Nordling et al. 2008). Additionally, ozone was estimated for the European cohorts using APMoSPHERE (Air Pollution Modelling for Support to Policy on Health and Environmental Risk in Europe) (Beelen et al. 2009), and for CAPPS/SAGE using inverse distance weighting of the closest three ambient monitors (within 50 km). NO_2_ was the only air pollution metric that was available across all six cohorts. PM_2.5_, PM_2.5_ absorbance, and ozone were used in sensitivity analyses because estimates of exposure to these pollutants were not available for all cohorts. Exposures were assigned for the first year of life based on geocoded birth addresses.

Candidate genes were selected based on availability of data across all six cohorts. The following single-nucleotide polymorphisms (SNPs) were investigated: *GSTP1* rs1138272 (merged with rs1799811; C→T: Ala^114^Val), *GSTP1* rs1695 (merged with rs947894; A→G: Ile^105^Val), and *TNF* rs1800629 (G→A: –308). Genotypic analyses were conducted at separate facilities using a variety of methods: The Canadian birth cohorts both used the Illumina BeadArray system (Illumina, San Diego, CA, USA) following the manufacturer’s protocol [CAPPS: peripheral blood was collected at 7 years (Carlsten et al. 2011a); SAGE: buccal samples were collected at 9 years (Kozyrskyj et al. 2009)]. The PIAMA study collected buccal swabs or blood at 4–8 years and used Competitive Allele-Specific PCR (polymerase chain reaction) using KASParTM genotyping chemistry (KBiosciences, Hoddesdon, UK). (Koopman et al. 2002). The German studies and BAMSE used matrix-assisted laser desorption/ionization–time of flight (MALDI-TOF) mass spectrometry (Sequenom, San Diego, CA, USA) following the manufacturer’s protocol [GINI/LISA: blood samples were collected at 6–10 years; BAMSE: blood samples were collected at 4 years (Melén et al. 2008)]. The German studies additionally used the restriction fragment length polymorphism approach for *GSTP1* rs1695 (Slama et al. 2010) and high-resolution melting-PCR using LightSNiP hybridization probe for *GSTP1* rs1138272 (TIB MolBiol, Berlin, Germany).

Data transfer and harmonization occurred in several stages. First, each cohort sent data dictionaries (translated to English, where applicable) to the main study center at the University of British Columbia. From each of the data dictionaries, a list of key variables was compiled and sent to each cohort to request data extraction. Data extracts were then prepared by each cohort and sent to the main study center for harmonization and merging. Data were collected at different time points across the cohorts, with differences in questionnaire wording and case definitions [see MacIntyre et al. (2013) for additional information]. In the pooled data set, new variables for this analysis were derived from data common to all cohorts.

*Statistical analysis*. We examined the main effect estimate for each SNP using dominant genetic models. Effect modification was examined in adjusted analyses of NO_2_ (per 10-μg/m^3^ increase) and asthma/wheeze, stratified by genotype. Interactions between genotype and NO_2_ were investigated using the log likelihood ratio for models with and without product interaction terms. Models were adjusted for potential confounders available across each cohort: study, city, infant sex, birth weight (< 2,500, 2,500–4,000, > 4,000 g), parental history of allergy (yes/no), maternal age at birth (≤ 24, 25–29, 30–34, ≥ 35 years), maternal smoking reported anytime during pregnancy (yes/no), environmental tobacco smoke reported in the home (yes/no), and intervention (yes/no). All pooled analyses used weighted logistic regression to adjust for inclusion of case–control data (Richardson et al. 2007). Sensitivity analyses investigated additional exposure metrics available for only a subset of pooled data [PM_2.5_ (per 4-μg/m^3^ increase), PM_2.5_ absorbance (per 0.5 10^–5^/m increase), and ozone (per 10-μg/m^3^ increase)). All *p*-values were corrected for multiple testing using Bonferroni adjustment, and adjusted *p*-values < 0.05 were considered statistically significant. All analyses used SAS version 9.2 (SAS Institute Inc., Cary, NC, USA).

## Results

Table 1 describes the population characteristics for children included in at least one analyses (*N*_total_ = 5,115). The population used for TAG had a lower prevalence of low birth weight (< 2,500 g) babies (3% vs. 12%), fewer mothers < 25 years of age (6% vs. 9%), more parents with reported atopy (55% vs. 47%), and fewer mothers who smoked during pregnancy (14% vs. 17%), compared to the total recruited population for each cohort.

**Table 1:** Population characteristics for the pooled TAG data set and for each individual cohort [*n* (%)].

Characteristic	Pooled	BAMSE	CAPPS	GINI	LISA	SAGE	PIAMA
Total population	5,115 (100)	912 (100)	346 (100)	1,126 (100)	697 (100)	178 (100)	1,856 (100)
Intervention	1,114 (21.8)	NA	181 (52.3)	528 (46.9)	NA	NA	405 (21.8)
Male sex	2,661 (52.0)	484 (53.1)	189 (54.6)	584 (51.9)	361 (51.8)	96 (53.9)	947 (51.0)
Birth weight (g)
< 2,500	150 (2.9)	45 (4.9)	16 (4.6)	19 (1.7)	0 (–)	13 (7.3)	57 (3.1)
2,500–4,000	4,085 (79.9)	706 (77.4)	256 (74.0)	952 (84.6)	587 (84.2)	132 (74.2)	1,452 (78.2)
> 4,000	880 (17.2)	161 (17.6)	74 (21.4)	155 (13.8)	110 (15.8)	33 (18.5)	347 (18.7)
Maternal smoking during pregnancy	688 (14.1)	119 (13.1)	29 (8.4)	149 (15.7)	91 (13.6)	18 (10.2)	282 (15.3)
Environmental tobacco smoke at home^*a*^	1,465 (28.6)	164 (18.0)	71 (20.5)	315 (28.0)	158 (22.7)	34 (19.1)	723 (39.0)
Parental atopy	2,802 (54.8)	530 (58.1)	319 (92.2)	459 (40.8)	348 (49.9)	126 (70.8)	1,020 (55.0)
Mother’s age at birth (years)
≤ 24	285 (5.6)	73 (8.0)	23 (6.7)	44 (3.9)	38 (5.5)	22 (12.4)	85 (4.5)
25–29	1,574 (30.8)	287 (31.5)	86 (24.9)	314 (27.9)	221 (31.7)	54 (30.3)	612 (33.0)
30–34	2,278 (44.5)	363 (40.2)	125 (36.1)	553 (49.1)	306 (44.1)	74 (41.6)	857 (46.2)
≥ 35	978 (19.1)	189 (20.7)	112 (32.4)	215 (19.1)	132 (18.9)	28 (15.7)	302 (16.3)
Current asthma	381 (7.5)	112 (12.3)	62 (17.9)	42 (3.7)	16 (2.3)	72 (40.5)	77 (4.2)
Ever asthma	878 (17.7)	272 (29.8)	144 (41.6)	86 (8.2)	30 (4.6)	72 (40.5)	274 (15.0)
Current wheeze	623 (12.5)	162 (17.9)	76 (22.0)	112 (10.1)	63 (9.4)	80 (69.0)	130 (7.0)
Ever wheeze	2,243 (44.9)	535 (58.7)	159 (46.0)	383 (35.2)	278 (40.0)	80 (69.0)	808 (44.0)
Ever asthma AND current wheeze	372 (7.6)	122 (13.5)	61 (17.3)	41 (3.9)	18 (2.8)	54 (46.6)	76 (4.2)
NA, not applicable. ^***a***^Defined as one or more person in the household regularly smoking indoors during the first year of life (exceptions: second year of life in GINI; eighth year of life in SAGE).							

A total of 5,115 children had complete data on asthma, wheeze, potential confounders, NO_2_, and at least one SNP (4,596 with *GSTP1* rs1138272, 4,748 with *GSTP1* rs1695, 4,483 with *TNF* rs1800629); 22% were part of an intervention, 52% were male, 29% were exposed to environmental tobacco smoke in the home, 55% had parental history of atopy, and 19% were born to mothers ≥ 35 years of age. Parents of 7.5% of the children reported current asthma (ranging from 2.3% to 40.5% in individual cohorts), 18% reported ever asthma (4.6–41.6%), 12.5% reported current wheeze (7.0–69.0%), 45% ever wheeze (35.2–69.0%), and 7.6% reported both ever asthma and current wheeze (“active asthma”; 2.8–46.6%).

Table 2 describes pollutant distributions for NO_2_, PM_2.5_, PM_2.5_ absorbance, and ozone. Data are presented for the pooled data set and by cohort or combinations of cohorts in which an identical LUR model was used in two different cohorts (Munich, Wesel, and Winnipeg). In general, the mean NO_2_ concentrations for Germany and the Netherlands were similar, whereas those for one of the Canadian locations (Winnipeg) and Sweden were lower. For the 2,743 children in sensitivity analyses, NO_2_ was correlated with PM_2.5_ absorbance (*r* = 0.72), weakly correlated with PM_2.5_ (*r* = 0.23), and inversely correlated with ozone (*r* = –0.19). The inverse correlation between NO_2_ and ozone was attributable to the atmospheric chemistry unique to each pollutant; and the weak correlation between PM_2.5_ and PM_2.5_ absorbance was likely attributable to differences in the composition and sources of air pollution in each region.

**Table 2:** Annual average pollutant statistics for the first year of life for pooled data and for each individual model.

Statistic	Pooled data	Sweden (BAMSE)	Munich, Germany (LISA/GINI)	Wesel and Leipzig, Germany (LISA/GINI)	The Netherlands (PIAMA)	Vancouver, Canada (CAPPS)	Winnipeg, Canada (CAPPS/SAGE)
NO_2_ (μg/m^3^)
Mean ± SD	22.7 ± 8.1	16.7 ± 9.1	27.5 ± 6.3	22.9 ± 3.5	24.6 ± 7.4	32.5 ± 6.0	12.2 ± 3.6
IQR	10.3	13.8	8.1	4.9	11.2	7.1	5.2
Range	2.2–66.8	2.2–49.9	19.5–66.9	13.9–41.4	12.6–58.4	18.9–55.1	4.1–21.5
Ozone (μg/m^3^)
Mean ± SD	36.8 ± 6.5	NA	43.9 ± 2.1	39.4 ± 3.6	33.8 ± 4.7	21.2 ± 2.8	NA
IQR	9.1	NA	1.0	6.9	4.3	4.8	NA
Range	13.3–55.9	NA	38.8–55.9	32.3–48.6	13.3–47.3	13.4–27.0	NA
PM_2.5_ (μg/m^3^)
Mean ± SD	15.2 ± 3.4	NA	13.3 ± 1.3	NA	16.7 ± 1.9	5.6 ± 2.6	NA
IQR	4.1	NA	1.5	NA	3.4	3.9	NA
Range	0.0–25.1	NA	11.9–21.9	NA	13.5–25.2	0.0–10.0	NA
PM_2.5_ absorbance (10^–5^/m)
Mean ± SD	1.7 ± 0.4	NA	1.7 ± 0.3	1.6 ± 0.2	1.7 ± 0.4	1.6 ± 1.2	NA
IQR	0.4	NA	0.3	0.2	0.6	1.1	NA
Range	0.0–5.0	NA	1.4–4.3	0.8–2.3	0.8–3.7	0.0–5.0	NA
Abbreviations: IQR, interquartile range; NA, not applicable.							

The population distribution and number of asthma cases by genotype are presented in Table 3. All SNPs satisfied Hardy–Weinberg equilibrium based on chi-square statistics (*p* < 0.05).

**Table 3:** Population distribution by minor allele carriers and homozygous major allele carriers for *GSTP1* and *TNF* (pooled data).

SNP genotype	No. of children	Current asthma cases [*n *(%)]	Children in the highest tertile of NO_2_ (*n*)	Children with current asthma in the highest tertile of NO_2_ [*n *(%)]
*GSTP1* rs1138272
TT/TC	789	80 (10.1)	224	15 (6.7)
CC	3,807	287 (7.5)	1,144	57 (5.0)
*GSTP1* rs1695
GG/GA	2,740	199 (7.3)	873	34 (3.9)
AA	2,008	170 (8.5)	618	39 (6.3)
*TNF* rs1800629
AA/AG	1,411	119 (8.4)	421	31 (7.4)
GG	3,072	244 (7.9)	917	40 (4.4)

*Main effect estimates*. Table 4 shows the main effect estimates for associations of the genotypes and air pollution exposures with the respiratory outcomes based on the pooled data for all cohorts combined. Effect estimates were calculated for homozygous and heterozygous minor allele carriers versus homozygous major allele carriers. The adjusted odds ratio (OR) for *GSTP1* rs1138272 and current asthma was 1.57 (95% CI: 1.16, 2.14; Bonferroni-corrected *p*-value = 0.012). Findings were similar for *GSTP1* rs1138272 and ever asthma (OR = 1.49; 95% CI: 1.20, 1.84; corrected *p*-value < 0.001) and current wheeze plus ever asthma (OR = 1.61; 95% CI: 1.22, 2.14; corrected *p*-value = 0.003). The adjusted OR for *GSTP1* rs1695 and current asthma was 0.87 (95% CI: 0.68, 1.12) in pooled analysis; associations between rs1695 and remaining respiratory outcomes were consistent with the null hypothesis. The adjusted OR for *TNF* rs1800629 and current asthma was 1.20 (95% CI: 0.92, 1.57). The association between ever wheeze and *TNF* rs1800629 was not statistically significant after adjusting for multiple testing (OR = 1.18; 95% CI: 1.02, 1.37; corrected *p*-value = 0.087). Statistically significant associations were estimated for NO_2_ and ever asthma (OR = 1.23; 95% CI: 1.03–1.46 per 10-μg/m^3^ increase; corrected *p*-value = 0.019) and for PM_2.5_ and current asthma (OR = 2.08; 95% CI: 1.35–3.19 per 4-μg/m^3^ increase; corrected *p*-value = 0.001).

**Table 4:** Main genetic and environmental effect estimates for *GSTP1*, *TNF*, and NO_2_ for asthma and wheeze at school age (pooled data).

SNP, exposure	Current asthma			Ever asthma			Current wheeze			Ever wheeze			Ever asthma and current wheeze		
	*n*	aOR^*a*^ (95% CI)	*p*^*b*^	*n*	aOR^*a*^ (95% CI)	*p*^*b*^	*n*	aOR^*a*^ (95% CI)	*p*^*b*^	*n*	aOR^*a*^ (95% CI)	*p*^*b*^	*n*	aOR^*a*^ (95% CI)	*p*^*b*^
*GSTP1* rs1138272 TT/TC v. CC	4,596	1.57 (1.16, 2.14)	0.012	4,465	1.49 (1.20, 1.84)	< 0.001	4,483	1.15 (0.84, 1.57)	0.560	4,487	1.01 (0.84, 1.22)	0.918	4,377	1.61 (1.22, 2.14)	0.003
*GSTP1* rs1695 GG/GA v. AA	4,748	0.87 (0.68, 1.12)	0.285	4,635	0.91 (0.77, 1.08)	0.430	4,659	1.02 (0.81, 1.29)	0.860	4,633	0.96 (0.83, 1.10)	0.803	4,547	0.94 (0.75, 1.19)	0.628
*TNF* rs1800629 AA/AG v. GG	4,483	1.20 (0.92, 1.57)	0.258	4,356	1.04 (0.87, 1.26)	0.647	4,371	1.22 (0.95, 1.56)	0.345	4,373	1.18 (1.02, 1.37)	0.087	4,267	1.10 (0.85, 1.41)	0.628
Traffic-related NO_2_ (per 10-μg/m^3^ increase)	5,115	1.14 (0.98, 1.48)	0.322	4,965	1.23 (1.03, 1.46)	0.019	4,999	1.12 (0.89, 1.40)	0.337	4,995	1.04 (0.91, 1.18)	0.610	4,874	1.13 (0.87, 1.46)	0.374
Ozone (per 10-μg/m^3^ increase)	2,743	0.81 (0.51, 1.27)	0.351	2,671	0.86 (0.66, 1.13)	0.277	2,728	1.29 (0.88, 1.88)	0.197	2,704	0.90 (0.74, 1.10)	0.318	2,659	1.01 (0.62, 1.67)	0.960
Traffic-related PM_2.5_ (per 4–μg/m^3^ increase)	2,743	2.08 (1.35, 3.19)	0.001	2,671	1.17 (0.86, 1.60)	0.323	2,728	1.33 (0.90, 1.95)	0.147	2,704	0.95 (0.74, 1.22)	0.687	2,659	1.46 (0.93, 2.32)	0.102
Traffic-related PM_2.5_ absorbance (per 0.5 10^–5^/m increase)	2,743	1.07 (0.91, 1.26)	0.441	2,671	1.06 (0.95, 1.19)	0.283	2,728	1.06 (0.93, 1.21)	0.395	2,704	1.00 (0.91, 1.10)	0.963	2,659	1.07 (0.92, 1.25)	0.369
aOR, adjusted odds ratio. ^***a***^Adjusted for study, city, intervention, infant sex, maternal age at birth, maternal smoking during pregnancy, environmental tobacco smoke in the home, birth weight, and parental atopy. ^***b***^*p*-Values were corrected for multiple testing using the Bonferroni method.															

*Genotype stratification and interaction*. The associations between NO_2_ during the first year of life and respiratory outcomes, stratified by genotype, and interaction *p*-values are presented in Table 5 for pooled data. Effect estimates were elevated and statistically significant in carriers of minor alleles of *GSTP1* rs1138272 and rs1695 in models for current asthma (corrected *p*-value for minor allele carriers = 0.006; corrected *p*-value for homozygous major allele carriers = 0.766; corrected *p*-values for interaction = 0.039) and ever asthma (corrected *p*-value for minor allele carriers = 0.048; corrected *p*-value for homozygous major allele carriers = 0.432; corrected *p*-values for interaction = 0.087), respectively. Overall, effect estimates were greater for minor allele carriers than for their respective homozygous major allele carriers in models for current asthma and ever asthma. Findings for *TNF* generally supported the null hypothesis.

**Table 5:** Association between traffic-related NO_2_ during the first year of life and asthma and wheeze at school age (pooled data).

Gene	Current asthma			Ever asthma			Current wheeze			Ever wheeze			Ever asthma and current wheeze		
	*n*	aOR^*a*^ (95% CI)	*p*^*b*^	*n*	aOR^*a*^ (95% CI)	*p*^*b*^	*n*	aOR^*a*^ (95% CI)	*p*^*b*^	*n*	aOR^*a*^ (95% CI)	*p*^*b*^	*n*	aOR^*a*^ (95% CI)	*p*^*b*^
*GSTP1* rs1138272
TT/TC	789	2.59 (1.43, 4.68)	0.006	769	1.64 (1.06, 2.53)	0.075	772	1.26 (0.71, 2.23)	0.821	775	1.17 (0.82, 1.65)	0.467	757	1.30 (0.67, 2.52)	0.669
CC	3,807	0.95 (0.68, 1.32)	0.766	3,696	1.20 (0.98, 1.48)	0.140	3,711	1.09 (0.82, 1.45)	0.821	3,712	1.09 (0.92, 1.29)	0.467	3,620	1.17 (0.85, 1.61)	0.669
Interaction^*c*^			0.039			0.012			0.096			0.024			0.180
*GSTP1* rs1695
GG/GA	2,704	1.43 (1.03, 1.98)	0.096	2,677	1.36 (1.08, 1.70)	0.048	2,695	1.18 (0.86, 1.60)	0.821	2,692	1.11 (0.93, 1.33)	0.467	2,632	1.16 (0.82, 1.65)	0.669
AA	2,008	0.82 (0.52, 1.32)	0.626	1,958	1.12 (0.84, 1.49)	0.432	1,964	1.03 (0.72, 1.46)	0.888	1,951	0.98 (0.79, 1.23)	0.886	1,915	1.09 (0.70, 1.68)	0.709
Interaction^*c*^			0.484			0.087			0.187			0.026			0.436
*TNF* rs1800629
AA/AG	1,411	1.30 (0.85, 2.00)	0.448	1,372	1.29 (0.94, 1.78)	0.143	1,383	1.04 (0.68, 1.59)	0.888	1,384	1.14 (0.86, 1.49)	0.467	1,350	1.13 (0.70, 1.82)	0.709
GG	3,072	1.07 (0.72, 1.57)	0.766	2,984	1.21 (0.97, 1.52)	0.140	2,988	1.24 (0.92, 1.68)	0.821	2,989	1.09 (0.91, 1.30)	0.467	2,917	1.15 (0.81, 1.63)	0.669
Interaction^*c*^			0.210			0.345			0.096			0.337			0.436
aOR, adjusted odds ratio. ^***a***^For a 10-μg/m^3^ increase in NO_2_. Adjusted for study, city, intervention, infant sex, maternal age at birth, maternal smoking during pregnancy, environmental tobacco smoke in the home, birth weight, and parental atopy. ^***b***^*p*-Values were corrected for multiple testing using the Bonferroni method. ^***c***^*p*-Values for interaction were calculated based on the likelihood-ratio tests between models with and without interaction terms and were corrected for multiple testing using the Bonferroni method.															

For children who were minor allele carriers for both *GSTP1* SNPs (rs1138272 and rs1695; *N*_total_ = 728) there were statistically significant associations between NO_2_ and current asthma (OR = 2.37; 95% CI: 1.31, 4.34 per 10 μg/m^3^ NO_2_; *p* = 0.005) and ever asthma (OR = 1.57; 95% CI: 1.04, 2.37; *p* = 0.032); analyses in remaining *GSTP1* rs1138272*rs1695 strata (rs1138272 TT/TC and rs1695 AA; rs1138272 CC and rs1695 GG/GA; rs1138272 CC and rs1695 AA) were nonsignificant (data not shown).

*Sensitivity analyses*. Sensitivity analyses are presented in the Supplemental Material, Tables S1–S12. The following cohorts (or cohort subsets) had assigned ozone, PM_2.5_, and PM_2.5_ absorbance; CAPPS Vancouver, LISA, GINI, and PIAMA. A total of 2,743 children had complete data on asthma, wheeze, potential confounders, NO_2_, ozone, PM_2.5_, PM_2.5_ absorbance, and at least one SNP of interest (*n* = 2,367 with *GSTP1* rs1138272; *n* = 2,559 with *GSTP1* rs1695; *n* = 2,318 with *TNF* rs1800629). Pooled estimates were recalculated for these additional air pollution metrics (see Supplemental Material, Tables S1–S3, respectively). In analyses stratified by genotype, there was an association between ozone and current wheeze (OR = 3.67; 95% CI: 1.05, 12.7; *N*_total_ = 405) in *GSTP1* rs1138272 minor allele carriers, and between PM_2.5_ and current asthma in all strata examined, although small sample size for some strata resulted in highly imprecise estimates. The results of multipollutant models examining NO_2_ and PM_2.5_, stratified by genotype, are presented in Supplemental Material, Table S4. NO_2_ effect estimates were sensitive to inclusion of PM_2.5_ in some analyses, but these results should be interpreted cautiously because data were available for only four cohorts.

The analyses applied to the pooled data were also applied to each individual cohort (see Supplemental Material, Tables S5–S10). Results showed some heterogeneity in the magnitude of effect estimate, but findings were generally not statistically significant due to small sample sizes. For example, the odds of current asthma per 10 μg/m^3^ NO_2_ for *GSTP1* rs1138272 minor allele carriers were 2.19 (0.54, 8.86) in BAMSE, 6.33 (95% CI: 0.66, 60.8) in GINI and LISA Munich, 5.44 (95% CI: 1.23, 24.0) in GINI and LISA Wesel, 2.27 (95% CI: 0.69, 7.49) in PIAMA, 2.30 (95% CI: 0.02, 256) in CAPPS Vancouver, and 19.20 (95% CI: 0.79, 466) in CAPPS and SAGE Winnipeg, versus 2.59 (95% CI: 1.43, 4.68) in pooled data. Further, the interactions between NO_2_ and genotype in pooled analyses were robust in reanalyses that sequentially excluded individual cohorts (data not shown).

Finally, analyses were rerun after restricting the pooled cohort to only those children in an observational (nonintervention) study arm (*N*_total_ = 3,695; see Supplemental Material, Table S11) and to children of Caucasian race (*N*_total_ = 4,821; see Supplemental Material, Table S12; data available only for CAPPS and SAGE). Results of these final sensitivities were in general agreement with findings in the full data set.

## Discussion

This study is the first Canadian–European consortium to examine the interaction between traffic-related air pollution and candidate genes of oxidative stress and inflammation in the development of childhood asthma. The pooled data set included 381 children with current asthma and 878 with ever asthma, and provided an unprecedented opportunity to identify populations genetically at risk for asthma development in relation to traffic-related air pollution. Our findings suggest that children with variant *GSTP1* genotypes may constitute a susceptible population at increased risk of asthma associated with air pollution.

The purpose of this work was to estimate the effect of two oxidative stress and inflammation genes on the relationship between traffic-related air pollution and the development of asthma in childhood. Asthma was associated with *GSTP1* rs1138272 in the study population. In analyses of NO_2_ and respiratory outcome, stratified by genotype, minor allele carriers for *GSTP1* rs1138272 and *GSTP1* rs1695 appeared to be more susceptible to the harmful effects of air pollution than their homozygous major allele–carrying counterparts. The interaction between *GSTP1* rs1138272 and NO_2_ was statistically significant in models for current asthma, ever asthma, and ever wheeze.

In this study we used data from six birth cohorts. Five of these cohorts have previously reported associations between traffic-related air pollution and respiratory outcomes during childhood (Carlsten et al. 2011b; Gehring et al. 2010; Morgenstern et al. 2008; Nordling et al. 2008); and three have previously reported gene–air pollution interactions in the development of childhood asthma (Carlsten et al. 2011a; Melén et al. 2008; Nordling et al. 2008). The BAMSE cohort reported a statistically significant association between NO_x_ and asthma at 4 years of age in children who were both minor allele carriers for *GSTP1* rs1695 and homozygous major allele carriers for *TNF* rs1800629 (OR = 2.9; 95% CI: 1.0–8.2 for a 44-μg/m^3^ increase in NO_x_) (Melén et al. 2008). We did not find strong evidence for a gene–gene–environment interaction in the pooled data set, although effect estimates were similar to those reported by Melén et al. (2008). The CAPPS study, which was particularly limited by sample size and did not have any homozygous minor allele carriers for *GSTP1* rs1138272, reported a negative but not statistically significant association between NO_2_ and asthma at 7 years of age in children who were heterozygous for *GSTP1* rs1138272 (OR = 0.76; 95% CI: 0.18–3.30 for a 7.2-μg/m^3^ increase in NO_2_; *N*_Total_ = 25) (Carlsten et al. 2011a). Finally, the PIAMA study has previously reported effect modification by interaction analyses for multiple *TLR2* and *TLR4* SNPs in the association between air pollution (using the same LUR models we presented) and childhood asthma in a subset of the PIAMA cohort (*N*_Total_ = 916; Kerkhof et al. 2010); but the current study is the first examination of *GSTP1* and *TNF*.

Polymorphisms in genes related to oxidative stress (*NQO1*, *GSTM1*, and *GSTP1*) have been associated with increased risk of asthma. Previous work has reported reductions in *GSTP1* expression among rs1138272 and rs1695 minor allele carriers (Moyer et al. 2008). Minor allele carriers of rs1695 were found to be at increased risk for asthma in numerous different study populations (Tamer et al. 2004), particularly when children were exposed to environmental tobacco smoke (Palmer et al. 2006) or living in regions with high air pollution (Romieu et al. 2006); and impaired lung function was observed in schoolchildren living in areas of high urban air pollution (Reddy et al. 2012). It has also been suggested that GST genes may modify the lung response after co-exposure to diesel exhaust and allergen (Gilliland 2009). Conversely, the rs1695 SNP has been negatively associated with asthma (Aynacioglu et al. 2004; Fryer et al. 2000) and was associated with a lower risk of incident asthma with exercise in communities with high (vs. low) levels of ozone (Islam et al. 2009). Few studies have had the opportunity to evaluate *GSTP1* rs1138272 and air pollution, partly because of the low frequency of minor allele carriers.

The inflammatory response gene *TNF* (rs1800629) has been reported to modify the relationship between ozone and asthma (Yang et al. 2008) and indoor home dampness and asthma (Tsai et al. 2011); however, similar to our results, no effect modification was noted in the association between traffic-related NO_2_ and adult-onset asthma (Castro-Giner et al. 2009).

In the present study we pooled data across six cohorts; but unfortunately, of the many SNPs associated with oxidative stress and inflammation, only three were typed across all cohorts. It is possible that this study was underpowered; however, homozygous and heterozygous minor allele carriers were combined into one category for all analyses (Melén et al. 2008; Salam et al. 2007), and three of the TAG cohorts intentionally increased the proportion of children with asthma or wheeze through study design.

NO_2_ is especially useful as a marker of within-city variability in exposure to traffic-related pollution and has been used as a marker of traffic pollution exposure in previous epidemiological investigations (Brunekreef 2007; Clark et al. 2010; Emenius et al. 2003; Gehring et al. 2010). Our results were not fully replicated in the subset (*n* = 2,743) of children with available data on additional markers of traffic-related (PM_2.5_ and PM_2.5_ absorbance) and regional (ozone) air pollution. The air pollution metrics examined here were considered only as markers for traffic-related air pollution; and the goal of this work was not to highlight the importance of one metric over another, but instead to highlight the significant potential impact of this air pollution source.

The TAG cohorts are unique in that they have individually assigned exposures with high spatial resolution for the first year of life based on residential address at birth—thus capturing the important exposure window during early life (Clark et al. 2010; Gehring et al. 2010). Although measurements for the LUR models were not originally taken during the first year of life, detailed investigations, including two conducted in our study areas, found the spatial distribution of traffic-related pollutants to be stable for up to 12 years (Eeftens et al. 2011; Gulliver et al. 2013; Wang et al. 2012). LUR model estimates were temporally adjusted using fixed-site monitoring data collected during the actual first year of life for cohorts that completed NO_2_ monitoring campaigns > 8 years after the birth year (CAPPS and SAGE).

An additional strength of the cohorts we included is that each recruited pregnant women or newborns and was therefore able to assess the development of asthma from birth. Ascertainment of physician-diagnosed asthma and wheeze symptoms via parental questionnaire is reasonably accurate (Burr 1992), particularly in countries with universal health care, where lack of insurance does not hinder access to health care. Further, the two cohorts with the highest prevalence of asthma (SAGE and CAPPS) also completed examinations by a pediatric allergist to confirm parent-reported asthma diagnoses. Further, although there were slight differences in questionnaire wording for asthma and predictors for air pollutant models, the general consistency in effect estimate direction and magnitude across analyses stratified by cohort or land use regression model (see Supplemental Material, Tables S5–S10) is reassuring and further supports our findings.

The findings presented here may have been confounded by exposures associated with oxidative stress or airway inflammation. We adjusted for environmental tobacco smoke and parental history of asthma or atopic disease, but it was not possible to adjust pooled analyses for the presence of allergens in the home or ethnicity. Smoking was associated with increased odds of disease in all pooled analyses; however, this finding was statistically significant only for “ever asthma” (data not shown). Sensitivity analyses excluding non-Caucasian children from the Canadian cohorts (< 80% Caucasian) were completed to ensure that our findings were not influenced by the ethnic composition of our population (Spielman et al. 2007); the proportion of Caucasian children in the European cohorts was very high (98%) and they were not restricted in the sensitivity. Finally, additional sensitivity analyses excluding children assigned to an intervention (Supplemental Material, Table S11; CAPPS, GINI, and PIAMA) were completed to ensure that our findings were not affected by preventive measures; this is particularly important because the CAPPS study has reported a protective effect (for children assigned to the intervention) on the development of childhood asthma up to 7 years of age (Chan-Yeung et al. 2000).

Our findings add to previous research by providing further insight into the complex relationship between genetics and environmental factors in the development of childhood asthma. We found the association between traffic-related air pollution and the development of childhood asthma to be influenced somewhat by *GSTP1* polymorphisms (rs1138272 and rs1695), and these findings support the hypothesis that the oxidative stress response is important in the pathogenesis of childhood asthma, particularly in the presence of air pollution. We found no indication of effect modification by the inflammatory response gene *TNF* (rs1800629) on the association between traffic-related air pollution and childhood asthma. *GSTP1* (rs1138272 and rs1695) minor allele carriers may be a susceptible population at increased risk of asthma in association with air pollution.

## Supplemental Material

(414 KB) PDF
