# Supplementary material for: GSTP1 and TNF Gene Variants and Associations between Air Pollution and Incident Childhood Asthma: The Traffic, Asthma and Genetics (TAG) Study
Source: Environ Health Perspect. 2014 Jan 24;122(4):418–24. doi: 10.1289/ehp.1307459 (PMC3984232; doi:10.1289/ehp.1307459)
Supplement: (414 KB) PDF [file ehp.1307459.s001.pdf]

Environmental Health Perspectives doi:10.1289/ehp.1307459

GSTP1 and *TNF* Gene Variants and Associations between Air Pollution and Incident Childhood Asthma: The Traffic, Asthma and Genetics (TAG) Study

Elaina A. MacIntyre, Michael Brauer, Erik Melén, Carl Peter Bauer, Mario Bauer, Dietrich Berdel, Anna Bergström, Bert Brunekreef, Moira Chan-Yeung, Claudia Klümper, Elaine Fuertes, Ulrike Gehring, Anna Gref, Joachim Heinrich, Olf Herbarth, Marjan Kerkhof, Gerard H. Koppelman, Anita L. Kozyrskyj, Göran Pershagen, Dirkje S. Postma, Elisabeth Thiering, Carla M.T. Tiesler, and Christopher Carlsten, for the TAG Study Group

Table of Contents

Appendix S1. Cohort Descriptions Page 3

References Page 6

Supplemental Material, Table S1. Association between ozone during the first year of life and asthma and wheeze at school age, stratified by genotype Page 7

Supplemental Material, Table S2. Association between traffic-related $PM_{2.5}$ during the first year of life and asthma and wheeze at school age, stratified by genotype Page 8

Supplemental Material, Table S3. Association between traffic-related $PM_{2.5}$ absorbance during the first year of life and asthma and wheeze at school age, stratified by genotype Page 9

Supplemental Material, Table S4. Multi-pollutant models (NO_2 and $PM_{2.5}$) for asthma and wheeze at school age, full dataset and stratified by genotype Page 10

Supplemental Material, Table S5. Main genetic and environmental effects for asthma and wheeze at school age; and association between traffic-related NO_2 and asthma and wheeze at school age, stratified by genotype (BAMSE) Page 11

Supplemental Material, Table S6. Main genetic and environmental effects for asthma and wheeze at school age; and association between traffic-related NO_2 and asthma and wheeze at school age, stratified by genotype (GINI & LISA – Munich) Page 12

Supplemental Material, Table S7. Main genetic and environmental effects for asthma and wheeze at school age; and association between traffic-related NO_2 and asthma and wheeze at school age, stratified by genotype (GINI & LISA – Wesel) Page 13

Supplemental Material, Table S8. Main genetic and environmental effects for asthma and wheeze at school age; and association between traffic-related NO_2 and asthma and wheeze at school age, stratified by genotype (PIAMA) Page 14

Supplemental Material, Table S9. Main genetic and environmental effects for asthma and wheeze at school age; and association between traffic-related NO_2 and asthma and wheeze at school age, stratified by genotype (CAPPS – Vancouver) Page 15

Supplemental Material, Table S10. Main genetic and environmental effects for asthma and wheeze at school age; and association between traffic-related NO_2 and asthma and wheeze at school age, stratified by genotype (CAPPS & SAGE – Winnipeg) Page 16

Supplemental Material, Table S11. Main genetic effects of *GSTP1* and *TNF* for asthma and wheeze at school age, for children without an intervention Page 17

Supplemental Material, Table S12. Main genetic and environmental effects for asthma and wheeze at school age; and association between traffic-related NO_2 and asthma and wheeze at school age, stratified by genotype (excluding non-Caucasian children) Page 18

Appendix S1. Cohort Descriptions

The Canadian Asthma Primary Prevention Study (CAPPS) is a prospective, randomized controlled study with follow-up to the age of 7 years. 545 high-risk infants were randomized prior to birth in the study centers of Vancouver and Winnipeg, Canada. High-risk was defined as having one first-degree relative with asthma or two first-degree relatives with other IgE mediated diseases. The multifaceted intervention included education and counseling on the risk factors of asthma, specifically dust mite and environmental tobacco smoke avoidance, and breastfeeding support. Parents completed questionnaires on respiratory symptoms and physician diagnoses at 1, 2 and 7 years. At 7 years children were examined by a pediatric allergist blinded to intervention status and questionnaire responses; and peripheral blood was obtained from children and their parents. Asthma was defined from questionnaires as at least two of more distinct episodes of cough (each lasting a minimum of 2 weeks), at least two distinct episodes of wheeze (each lasting a minimum of 1 week), plus at least one of the following: nocturnal cough at least once per week (in absence of a cold), hyperpnoea-induced cough or wheeze at any time, or response to treatment with β-agonist and/or anti-inflammatory drugs (Chan-Yeung et al. 2000; Carlsten et al. 2011).

The Study of Asthma, Genetics and Environment (SAGE) is a population-based birth cohort. Children were identified for inclusion from a provincial healthcare registry. The study included all 13,980 children born in the province of Manitoba in 1995 with continued residence in the province through 2002. Surveys were sent to each family when children were 7 years old and, from the 3,598 responders, 723 children were selected for a nested case-control study of asthma (246 asthmatics; 477 controls). Children living in rural areas, low-income neighborhoods and First Nations communities

were over-sampled. At mean age of 9 years, children were examined by a pediatric allergist for allergic diseases, including asthma, and symptoms (Kozyrskyj et al. 2009).

The Children, Allergy, Milieu, Stockholm, Epidemiological Survey (BAMSE) is a population based prospective birth cohort study with follow-up through the age of 16. Between February 1994 and November 1996 newborns were recruited at their first child health visit in predefined areas of Stockholm, Sweden (n = 4,089). Infants were excluded if their family was planning to move during the first year of life, an older sibling was already enrolled, serious illness during the neonatal period or parents had insufficient knowledge of Swedish. Parental questionnaires were used to assess physician diagnosed asthma, allergic rhinitis and eczema; and episodes of wheezing at ages 1, 2, 4 and 8 years (Wickman et al. 2002). At 4 years of age 2,298 children provided blood samples and a sub-sample of this group was used to populate a nested case-control study of wheeze (497 wheezers; 485 randomly selected controls) (Melén et al. 2008).

The German infant study on the influence of nutrition intervention plus environmental and genetic influences on allergy development (GINIplus) is a population based prospective birth cohort, with an intervention component and follow-up to the age of 15 years. Between September 1995-June 1998 parents attending one of 18 maternity hospitals in the cities of Munich or Wesel were invited to participate. A total of 5,991 healthy full-term newborns whose parents were fluent in German were recruited. A subgroup of 2,252 infants with at least one atopic parent or sibling were assigned to the intervention group and randomly allocated to one of four study formulas if their parents chose not to breastfeed. Parental questionnaires were used to assess physician diagnosed asthma, allergic rhinitis and eczema; and episodes of wheezing at ages 1, 2, 3, 4, 6 and 10 years (Gehring et al. 2002). Clinical examinations and blood samples for DNA extraction were obtained at 6 and 10 years.

The influence of life style factors on the development of the immune system and allergies in East and West Germany plus the influence of traffic emissions and genetics (LISAplus) study is a population based prospective birth cohort study with follow-up to the age of 15 years. Between December 1997-January 1999 parents attending one of 14 obstetrical clinics or hospitals throughout the cities of Munich, Leipzig, Wesel or Bad Honnef were invited to participate. A total of 3,095 healthy full term newborns whose parents were born in Germany and had German citizenship were recruited. Parental questionnaires were used to assess physician diagnosed asthma, allergic rhinitis and eczema; and episodes of wheezing at ages 0.5, 1, 1.5, 2, 4, 6 and 10 years (Gehring et al. 2002). Clinical examinations and blood samples for DNA extraction were obtained at 6 and 10 years.

The Prevention and Incidence of Asthma and Mite Allergy (PIAMA) study is a population based prospective birth cohort study, with an intervention component, and follow-up through the age of 15 years. Between May 1996 – December 1997, 3963 children were born to mothers who had been recruited during their first trimester of pregnancy from midwife practices in three different regions of The Netherlands. Children were divided into high- and low-risk groups based on a screening questionnaire on allergic disease of their mother. Children in the high-risk group were initially assigned to the intervention arm (n = 855) with a random subset allocated to the natural history arm (n = 472) with low-risk children. The intervention required use of a mite-impermeable mattress and pillow cover (Koopman et al. 2002). Information on physician-diagnosed asthma, allergic rhinitis and eczema; and episodes of wheezing were ascertained through parental questionnaires completed at each birthday until 8 years. Blood samples were collected at 4, 8, 11 and 12 years.

References

Carlsten C, Dybuncio A, Becker A, Chan-Yeung M, Brauer M. 2011 Traffic-related air pollution and incident asthma in a high-risk birth cohort. Occup Environ Med. Apr;68(4):291-295.

Chan-Yeung M, Manfreda J, Dimich-Ward H, Ferguson A, Watson W, Becker A. 2000 A randomized controlled study on the effectiveness of a multifaceted intervention program in the primary prevention of asthma in high-risk infants. Arch Pediatr Adolesc Med. Jul;154(7):657-663.

Gehring U, Cyrys J, Sedlmeir G, Brunekreef B, Bellander T, Fischer P, et al. 2002 Traffic-related air pollution and respiratory health during the first 2 yrs of life. Eur Respir J. Apr;19(4):690-698.

Koopman LP, van Strien RT, Kerkhof M, Wijga A, Smit HA, de Jongste JC, et al. 2002 Placebo-controlled trial of house dust mite-impermeable mattress covers: effect on symptoms in early childhood. Am J Respir Crit Care Med. Aug 1;166(3):307-313.

Kozyrskyj AL, HayGlass KT, Sandford AJ, Pare PD, Chan-Yeung M, Becker AB. 2009 A novel study design to investigate the early-life origins of asthma in children (SAGE study). Allergy. Aug;64(8):1185-1193.

Melén E, Nyberg F, Lindgren CM, Berglind N, Zucchelli M, Nordling E, et al. 2008 Interactions between glutathione S-transferase P1, tumor necrosis factor, and traffic-related air pollution for development of childhood allergic disease. Environ Health Perspect. Aug;116(8):1077-1084.

Wickman M, Kull I, Pershagen G, Nordvall SL. 2002The BAMSE project: presentation of a prospective longitudinal birth cohort study. Pediatr Allergy Immunol.;13 Suppl 15:11-13.

Supplemental Material, Table S1. Association between ozone during the first year of life and asthma and wheeze at school age, stratified by genotype (pooled data, n = 2,743).

**Genotype**	**Current asthma**		**Ever asthma**		**Current wheeze**		**Ever wheeze**		**Ever asthma and current wheeze**	
	**N**	**aOR[a] (95%CI)**	**N**	**aOR[a] (95%CI)**	**N**	**aOR[a] (95%CI)**	**N**	**aOR[a] (95%CI)**	**N**	**aOR[a] (95%CI)**
***GSTP1* rs1138272**										
TT/TC	406	1.08(0.44, 2.63)	402	1.13(0.57, 2.21)	405	3.67(1.05, 12.7)	405	1.37(0.79, 2.37)	401	6.92(1.82, 26.5)
CC	1961	0.77(0.47, 1.26)	1910	0.84(0.62, 1.12)	1948	1.10(0.75, 1.63)	1933	0.84(0.67, 1.04)	1900	0.66(0.41, 1.06)
***GSTP1* rs1695**										
GG/GA	1517	0.79(0.43, 1.46)	1485	0.89(0.62, 1.28)	1512	1.59(0.93, 2.73)	1500	0.84(0.65, 1.09)	1480	1.20(0.56, 2.60)
AA	1042	0.72(0.36, 1.43)	1014	0.73(0.48, 1.12)	1037	1.12(0.62, 2.02)	1024	0.90(0.65, 1.26)	1009	0.84(0.42, 1.72)
***TNF* rs1800629**										
AA/AG	763	0.76(0.35, 1.66)	748	0.77(0.49, 1.19)	758	1.34(0.67, 2.70)	757	0.95(0.67, 1.35)	744	1.11(0.41, 2.97)
GG	1555	0.90(0.52, 1.54)	1516	0.91(0.64, 1.29)	1546	1.28(0.80, 2.03)	1532	0.84(0.65, 1.08)	1509	0.95(0.53, 1.70)

[a]For a 10 $\mu g/m^3$ increase in O_3. Adjusted for study, city, intervention, gender, maternal age at birth, maternal smoking during pregnancy, environmental tobacco smoke in the home, birth weight and parental atopy.

Supplemental Material, Table S2. Association between traffic-related $PM_{2.5}$ during the first year of life and asthma and wheeze at school age, stratified by genotype (pooled data, n = 2,743).

Genotype	Current asthma		Ever asthma		Current wheeze		Ever wheeze		Ever asthma and current wheeze	
	N	aOR[a] (95%CI)	N	aOR[a] (95%CI)	N	aOR[a] (95%CI)	N	aOR[a] (95%CI)	N	aOR[a] (95%CI)
***GSTP1* rs1138272**										
TT/TC	406	7.75(2.52, 23.9)	402	1.68(0.73, 3.85)	405	0.90(0.28, 2.92)	405	1.00(0.51, 1.97)	401	2.13(0.58, 7.78)
CC	1961	1.90(1.13, 3.21)	1910	1.09(0.76, 1.57)	1948	1.55(0.98, 2.47)	1933	1.00(0.73, 1.36)	1900	1.61(0.94, 2.78)
***GSTP1* rs1695**										
GG/GA	1517	2.17(1.20, 3.94)	1485	1.12(0.73, 1.71)	1512	1.13(0.62, 2.09)	1500	1.05(0.75, 1.48)	1480	1.06(0.52, 2.16)
AA	1042	2.09(1.09, 4.00)	1014	1.37(0.85, 2.21)	1037	1.66(0.98, 2.80)	1024	0.95(0.63, 1.44)	1009	1.96(1.03, 3.73)
***TNF* rs1800629**										
AA/AG	763	1.78(0.72, 4.37)	748	1.09(0.54, 2.19)	758	1.21(0.50, 2.92)	757	1.09(0.63, 1.88)	744	1.34(0.48, 3.75)
GG	1555	2.41(1.31, 4.42)	1516	1.22(0.84, 1.79)	1546	1.62(0.98, 2.68)	1532	1.01(0.73, 1.40)	1509	1.71(0.97, 3.01)

[a]For a 4 $\mu g/m^3$ increase in $PM_{2.5}$. Adjusted for study, city, intervention, gender, maternal age at birth, maternal smoking during pregnancy, environmental tobacco smoke in the home, birth weight and parental atopy.

Supplemental Material, Table S3. Association between traffic-related $PM_{2.5}$ absorbance during the first year of life and asthma and wheeze at school age, stratified by genotype (pooled data, n = 2,743).

Genotype	Current asthma		Ever asthma		Current wheeze		Ever wheeze		Ever asthma and current wheeze	
	**N**	**aOR[a] (95%CI)**	**N**	**aOR[a] (95%CI)**	**N**	**aOR[a] (95%CI)**	**N**	**aOR[a] (95%CI)**	**N**	**aOR[a] (95%CI)**
***GSTP1* rs1138272**										
TT/TC	406	2.10(0.86, 5.12)	402	1.17(0.85, 1.62)	405	1.38(0.92, 2.06)	405	1.08(0.82, 1.44)	401	1.55(0.80, 3.00)
CC	1961	1.03(0.86, 1.25)	1910	1.05(0.93, 1.19)	1948	1.04(0.90, 1.21)	1933	1.02(0.91, 1.14)	1900	1.08(0.91, 1.27)
***GSTP1* rs1695**										
GG/GA	1517	1.23(0.98, 1.55)	1485	1.13(0.98, 1.31)	1512	1.17(0.97, 1.40)	1500	1.07(0.94, 1.21)	1480	1.13(0.91, 1.41)
AA	1042	0.99(0.75, 1.29)	1014	1.02(0.86, 1.21)	1037	1.02(0.84, 1.24)	1024	0.98(0.84, 1.15)	1009	1.08(0.87, 1.34)
***TNF* rs1800629**										
AA/AG	763	1.09(0.81, 1.48)	748	1.13(0.90, 1.42)	758	1.11(0.85, 1.45)	757	1.04(0.85, 1.27)	744	1.05(0.77, 1.42)
GG	1555	1.02(0.81, 1.27)	1516	1.03(0.90, 1.18)	1546	1.05(0.90, 1.23)	1532	1.02(0.90, 1.14)	1509	1.08(0.90, 1.30)

[a]For a 0.5 10^{-5}/m increase in $PM_{2.5}$ absorbance. Adjusted for study, city, intervention, gender, maternal age at birth, maternal smoking during pregnancy, environmental tobacco smoke in the home, birth weight and parental atopy.

Supplemental Material, Table S4. Multi-pollutant models (NO_2 and $PM_{2.5}$) for asthma and wheeze at school age, full dataset and stratified by genotype (pooled[a] data, n = 2,755).

Genotype	Pollutant	Current asthma		Ever asthma		Current wheeze		Ever wheeze		Ever asthma and current wheeze	
		aOR[b] (95%CI)	p-val	aOR[b] (95%CI)	p-val	aOR[b] (95%CI)	p-val	aOR[b] (95%CI)	p-val	aOR[b] (95%CI)	p-val
**Complete Data**											
	NO_2	0.96(0.90, 1.03)	0.223	1.00(0.96, 1.04)	0.976	0.97(0.92, 1.02)	0.222	1.00(0.96, 1.03)	0.841	0.96(0.90, 1.03)	0.243
	$PM_{2.5}$	1.35(1.07, 1.70)	0.012	1.03(0.89, 1.20)	0.669	1.18(0.98, 1.43)	0.081	1.00(0.87, 1.14)	0.945	1.22(0.98, 1.52)	0.071
***GSTP1* rs1138272**											
TT/TC	NO_2	0.91(0.73, 1.13)	0.380	1.03(0.91, 1.15)	0.683	0.85(0.71, 1.01)	0.072	0.96(0.86, 1.07)	0.486	0.84(0.69, 1.02)	0.083
TT/TC	$PM_{2.5}$	2.19(1.03, 4.65)	0.041	1.03(0.67, 1.60)	0.887	1.56(0.90, 2.72)	0.114	1.14(0.75, 1.74)	0.533	1.95(1.09, 3.50)	0.025
CC	NO_2	0.96(0.89, 1.04)	0.335	1.00(0.95, 1.04)	0.846	0.99(0.94, 1.04)	0.727	1.01(0.97, 1.05)	0.657	0.99(0.92, 1.06)	0.814
CC	$PM_{2.5}$	1.29(1.01, 1.65)	0.041	1.02(0.87, 1.21)	0.774	1.15(0.94, 1.41)	0.174	0.97(0.83, 1.12)	0.660	1.15(0.91, 1.46)	0.236
***GSTP1* rs1695**											
GG/GA	NO_2	1.00(0.89, 1.13)	0.947	1.01(0.95, 1.08)	0.658	0.97(0.90, 1.05)	0.471	1.01(0.96, 1.06)	0.804	0.96(0.85, 1.08)	0.456
GG/GA	$PM_{2.5}$	1.19(0.76, 1.85)	0.446	0.97(0.77, 1.23)	0.815	1.14(0.85, 1.54)	0.386	0.98(0.80, 1.21)	0.877	1.17(0.80, 1.72)	0.417
AA	NO_2	0.93(0.85, 1.02)	0.125	0.99(0.94, 1.05)	0.864	0.98(0.92, 1.04)	0.498	0.990.94, 1.04)	0.655	0.99(0.92, 1.06)	0.715
AA	$PM_{2.5}$	1.40(1.06, 1.84)	0.017	1.09(0.89, 1.33)	0.412	1.20(0.96, 1.52)	0.116	1.02(0.84, 1.24)	0.816	1.22(0.95, 1.56)	0.112
***TNF* rs1800629**											
AA/AG	NO_2	0.95(0.84, 1.07)	0.406	0.98(0.90, 1.07)	0.693	0.95(0.86, 1.04)	0.266	1.00(0.93, 1.07)	0.917	0.93(0.81, 1.06)	0.292
AA/AG	$PM_{2.5}$	1.34(0.87, 2.05)	0.184	1.07(0.77, 1.48)	0.687	1.26(0.86, 1.85)	0.232	1.04(0.77, 1.39)	0.810	1.32(0.89, 1.95)	0.170
GG	NO_2	0.95(0.87, 1.04)	0.286	1.01(0.96, 1.06)	0.819	0.99(0.93, 1.05)	0.650	1.00(0.96, 1.04)	0.940	0.97(0.90, 1.05)	0.425
GG	$PM_{2.5}$	1.42(1.04, 1.93)	0.026	1.03(0.86, 1.23)	0.784	1.17(0.93, 1.47)	0.171	0.99(0.84, 1.16)	0.906	1.24(0.94, 1.63)	0.124

[a]Including CAPPS Vancouver, GINI, LISA & PIAMA. [b]For a 1-unit increase in pollutant. Adjusted for study, city, intervention, gender, maternal age at birth, maternal smoking during pregnancy, environmental tobacco smoke in the home, birth weight and parental atopy.

Supplemental Material, Table S5. Main genetic and environmental effects for asthma and wheeze at school age; and association between traffic-related NO_2 and asthma and wheeze at school age, stratified by genotype (BAMSE, n = 912).

Model	Current asthma			Ever asthma			Current wheeze			Ever wheeze			Ever asthma and current wheeze		
	N	aOR[a] (95%CI)	p-val[b]	N	aOR (95%CI)	p-val[b]	N	aOR (95%CI)	p-val[b]	N	aOR (95%CI)	p-val[b]	N	aOR (95%CI)	p-val[b]
Main Effects															
GSTP1 rs1138272 TT/TC v. CC	856	1.53(0.83, 2.83)	0.51	856	1.66(1.06, 2.6)	0.078	850	1.35(0.85, 2.14)	0.302	856	1.24(0.84, 1.82)	0.429	850	1.35(0.82, 2.23)	0.356
GSTP1 rs1695 GG/GA v. AA	891	0.85(0.52, 1.38)	0.758	891	0.89(0.63, 1.25)	0.488	885	1.19(0.83, 1.7)	0.351	891	1.00(0.76, 1.32)	0.993	885	0.97(0.65, 1.45)	0.891
TNF rs1800629 AA/AG v. GG	849	1.09(0.64, 1.85)	0.758	849	1.26(0.86, 1.85)	0.348	843	1.33(0.90, 1.95)	0.302	849	1.23(0.90, 1.69)	0.429	843	1.32(0.86, 2.04)	0.356
Traffic-related NO_2 (per $10\mu g/m^3$)	912	1.04(0.61, 1.75)	0.896	912	1.49(1.00, 2.22)	0.05	906	1.28(0.82, 2.01)	0.277	912	1.45(1.04, 2)	0.026	906	1.18(0.69, 2)	0.552
Stratified Results															
***GSTP1* rs1138272**															
TT/TC	142	2.19(0.54, 8.86)	0.921	142	1.71(0.54, 5.36)	0.432	142	2.25(0.60, 8.4)	0.456	142	1.34(0.59, 3.08)	0.487	142	1.44(0.22, 9.32)	0.986
CC	714	0.95(0.51, 1.78)	0.921	714	1.74(1.10, 2.77)	0.057	708	1.13(0.68, 1.89)	0.776	714	1.64(1.13, 2.38)	0.054	708	1.26(0.68, 2.34)	0.986
***GSTP1* rs1695**															
GG/GA	487	1.07(0.50, 2.28)	0.921	487	1.88(1.11, 3.19)	0.057	486	1.47(0.84, 2.57)	0.456	487	1.61(1.01, 2.58)	0.136	486	1.21(0.60, 2.45)	0.986
AA	404	0.95(0.42, 2.16)	0.921	404	1.14(0.64, 2.02)	0.654	399	0.93(0.45, 1.95)	0.855	404	1.28(0.78, 2.11)	0.389	399	0.99(0.42, 2.37)	0.991
***TNF* rs1800629**															
AA/AG	242	1.16(0.43, 3.13)	0.921	242	1.71(0.85, 3.44)	0.262	240	2.00(0.83, 4.82)	0.456	242	1.62(0.73, 3.59)	0.353	240	1.44(0.61, 3.44)	0.986
GG	607	0.96(0.40, 2.28)	0.921	607	1.39(0.81, 2.37)	0.353	603	1.16(0.61, 2.21)	0.776	607	1.40(0.98, 2.02)	0.136	603	1.10(0.49, 2.43)	0.986

[a]Adjusted for study, city, intervention, gender, maternal age at birth, maternal smoking during pregnancy, environmental tobacco smoke in the home, birth weight and parental atopy. [b]P-values were corrected for multiple testing using the Bonferroni method.

Supplemental Material, Table S6. Main genetic and environmental effects for asthma and wheeze at school age; and association between traffic-related NO_2 and asthma and wheeze at school age, stratified by genotype (GINI & LISA – Munich only, n = 726).

Model	Current asthma			Ever asthma			Current wheeze			Ever wheeze			Ever asthma and current wheeze		
	N	aOR[a] (95%CI)	p-val[b]	N	aOR (95%CI)	p-val[b]	N	aOR (95%CI)	p-val[b]	N	aOR (95%CI)	p-val[b]	N	aOR (95%CI)	p-val[b]
Main Effects															
GSTP1 rs1138272 TT/TC v. CC	385	2.53(0.64, 10)	0.300	360	1.73(0.55, 5.5)	0.873	380	0.82(0.30, 2.28)	0.705	375	1.06(0.60, 1.88)	0.84	358	3.57(0.85, 15)	0.243
GSTP1 rs1695 GG/GA v. AA	596	0.81(0.26, 2.54)	0.715	566	1.07(0.45, 2.57)	0.873	594	0.85(0.47, 1.57)	0.705	580	1.09(0.76, 1.57)	0.840	564	1.42(0.41, 4.89)	0.764
TNF rs1800629 AA/AG v. GG	357	2.28(0.65, 8.01)	0.300	333	0.83(0.26, 2.71)	0.873	352	1.82(0.77, 4.3)	0.519	347	1.36(0.82, 2.26)	0.708	331	1.29(0.24, 6.97)	0.764
Traffic-related NO_2 (per 10µg/m^3)	726	1.18(0.71, 1.98)	0.522	684	1.01(0.64, 1.58)	0.973	720	1.00(0.66, 1.51)	0.991	706	0.91(0.70, 1.19)	0.504	681	0.90(0.44, 1.85)	0.776
Stratified Results															
***GSTP1* rs1138272**															
TT/TC	64	6.33(0.66, 60.8)	0.633	62	113(0.22, 58E3)	0.828	64	1.43(0.26, 7.9)	0.982	64	1.28(0.36, 4.59)	0.977	62	6.33(0.66, 61.1)	0.222
CC	321	1.09(0.43, 2.74)	0.858	298	0.76(0.35, 1.65)	0.893	316	0.87(0.46, 1.66)	0.982	311	0.98(0.64, 1.51)	0.977	296	0.29(0.11, 0.74)	0.060
***GSTP1* rs1695**															
GG/GA	361	1.25(0.55, 2.84)	0.712	342	0.97(0.44, 2.16)	0.941	360	0.99(0.46, 2.13)	0.982	352	0.94(0.63, 1.39)	0.977	341	0.78(0.21, 2.99)	0.823
AA	235	0.55(0.22, 1.4)	0.633	224	0.70(0.34, 1.44)	0.893	234	1.07(0.63, 1.82)	0.982	228	0.95(0.61, 1.46)	0.977	223	1.11(0.45, 2.74)	0.823
***TNF* rs1800629**															
AA/AG	101	1.46(0.37, 5.78)	0.712	93	1.46(0.36, 5.82)	0.893	99	1.10(0.29, 4.14)	0.982	99	0.99(0.43, 2.26)	0.977	92	0.02(0.00, 1.45)	0.222
GG	256	1.42(0.57, 3.54)	0.712	240	1.04(0.49, 2.24)	0.941	253	0.96(0.57, 1.63)	0.982	248	0.96(0.59, 1.57)	0.977	239	1.14(0.44, 2.98)	0.823

[a]Adjusted for study, city, intervention, gender, maternal age at birth, maternal smoking during pregnancy, environmental tobacco smoke in the home, birth weight and parental atopy. [b]P-values were corrected for multiple testing using the Bonferroni method.

Supplemental Material, Table S7. Main genetic and environmental effects for asthma and wheeze at school age; and association between traffic-related NO_2 and asthma and wheeze at school age, stratified by genotype (GINI & LISA – Wesel only, n =1,097).

**Model**	**Current asthma**			**Ever asthma**			**Current wheeze**			**Ever wheeze**			**Ever asthma and current wheeze**		
	**N**	**aOR[a] (95%CI)**	**p-val[b]**	**N**	**aOR[a] (95%CI)**	**p-val[b]**	**N**	**aOR[a] (95%CI)**	**p-val[b]**	**N**	**aOR[a] (95%CI)**	**p-val[b]**	**N**	**aOR[a] (95%CI)**	**p-val[b]**
Main Effects															
GSTP1 rs1138272 TT/TC v. CC	1014	1.11(0.50, 2.46)	0.803	940	1.13(0.64, 1.99)	0.667	982	1.76(1.12, 2.77)	0.045	997	1.39(1.00, 1.94)	0.147	930	1.69(0.84, 3.39)	0.420
GSTP1 rs1695 GG/GA v. AA	940	0.76(0.39, 1.47)	0.617	889	0.82(0.51, 1.33)	0.635	928	1.10(0.71, 1.71)	0.655	923	1.20(0.91, 1.57)	0.293	878	1.21(0.60, 2.45)	0.618
TNF rs1800629 AA/AG v. GG	953	0.68(0.33, 1.42)	0.617	882	0.70(0.41, 1.2)	0.588	923	1.28(0.84, 1.94)	0.378	936	1.06(0.79, 1.41)	0.7	872	0.84(0.41, 1.69)	0.618
Traffic-related NO_2 (per 10µg/m^3)	1097	1.21(0.52, 2.81)	0.656	1021	1.59(0.89, 2.85)	0.118	1064	0.82(0.47, 1.44)	0.495	1080	1.15(0.78, 1.7)	0.487	1010	1.56(0.70, 3.46)	0.273
Stratified Results															
***GSTP1* rs1138272**															
TT/TC	187	5.44(1.23, 24)	0.075	171	2.98(0.86, 10.4)	0.230	180	0.73(0.27, 1.96)	0.938	183	1.18(0.47, 2.97)	0.726	169	2.58(0.63, 10.6)	0.561
CC	827	0.86(0.30, 2.49)	0.787	769	1.30(0.63, 2.67)	0.584	802	0.89(0.45, 1.76)	0.938	814	1.35(0.84, 2.16)	0.434	761	1.30(0.51, 3.29)	0.694
***GSTP1* rs1695**															
GG/GA	541	1.99(0.67, 5.94)	0.436	512	1.87(0.86, 4.06)	0.230	533	0.58(0.26, 1.31)	0.618	533	0.90(0.52, 1.56)	0.726	504	1.63(0.54, 4.93)	0.672
AA	399	0.77(0.21, 2.83)	0.787	377	1.45(0.51, 4.13)	0.584	395	1.04(0.39, 2.78)	0.938	390	1.65(0.83, 3.26)	0.434	374	1.70(0.43, 6.73)	0.672
***TNF* rs1800629**															
AA/AG	307	5.69(1.45, 22.4)	0.075	285	2.77(0.99, 7.78)	0.230	299	0.54(0.21, 1.4)	0.618	301	1.65(0.78, 3.48)	0.434	282	2.48(1.04, 5.91)	0.240
GG	646	0.74(0.22, 2.45)	0.787	597	1.21(0.56, 2.63)	0.628	624	1.04(0.50, 2.13)	0.938	635	1.29(0.77, 2.17)	0.497	590	1.07(0.32, 3.54)	0.911

[a]Adjusted for study, city, intervention, gender, maternal age at birth, maternal smoking during pregnancy, environmental tobacco smoke in the home, birth weight and parental atopy. [b]P-values were corrected for multiple testing using the Bonferroni method.

Supplemental Material, Table S8. Main genetic and environmental effects for asthma and wheeze at school age; and association between traffic-related NO_2 and asthma and wheeze at school age, stratified by genotype (PIAMA, n = 1,387).

Model	Current asthma			Ever asthma			Current wheeze			Ever wheeze			Ever asthma and current wheeze		
	N	aOR[a] (95%CI)	p-val[b]	N	aOR[a] (95%CI)	p-val[b]	N	aOR[a] (95%CI)	p-val[b]	N	aOR[a] (95%CI)	p-val[b]	N	aOR[a] (95%CI)	p-val[b]
Main Effects															
GSTP1 rs1138272 TT/TC v. CC	1360	1.81(1.00, 3.26)	0.150	1336	1.47(1.02, 2.11)	0.111	1351	1.35(0.81, 2.26)	0.734	1344	1.08(0.81, 1.43)	0.617	1327	1.92(1.06, 3.47)	0.090
GSTP1 rs1695 GG/GA v. AA	1346	0.73(0.44, 1.23)	0.241	1322	0.91(0.67, 1.25)	0.676	1338	0.90(0.59, 1.38)	0.734	1330	0.83(0.66, 1.04)	0.150	1314	0.82(0.48, 1.4)	0.699
TNF rs1800629 AA/AG v. GG	1344	1.55(0.92, 2.62)	0.153	1320	0.93(0.68, 1.29)	0.676	1335	1.08(0.70, 1.67)	0.734	1328	1.24(0.98, 1.56)	0.150	1311	0.95(0.55, 1.65)	0.857
Traffic-related NO_2 (per $10 \mu g/m^3$)	1387	1.48(0.76, 2.87)	0.248	1363	1.15(0.76, 1.75)	0.513	1378	1.25(0.71, 2.22)	0.44	1371	0.98(0.71, 1.35)	0.908	1354	1.36(0.69, 2.67)	0.369
Stratified Results															
GSTP1 **rs1138272**															
TT/TC	245	2.27(0.69, 7.49)	0.360	244	1.16(0.43, 3.14)	0.889	244	0.40(0.04, 3.9)	0.651	244	0.80(0.36, 1.79)	0.931	243	0.43(0.04, 5.04)	0.734
CC	1115	1.41(0.64, 3.07)	0.588	1092	1.24(0.78, 1.98)	0.830	1107	1.57(0.87, 2.83)	0.264	1100	1.06(0.74, 1.52)	0.931	1084	1.88(0.95, 3.74)	0.190
GSTP1 **rs1695**															
GG/GA	797	1.08(0.45, 2.6)	0.974	787	0.86(0.48, 1.52)	0.889	793	0.98(0.42, 2.27)	0.966	791	1.17(0.77, 1.77)	0.931	783	0.75(0.25, 2.29)	0.734
AA	549	2.41(0.92, 6.34)	0.360	535	2.11(1.13, 3.96)	0.114	545	1.95(0.85, 4.51)	0.264	539	0.90(0.53, 1.55)	0.931	531	2.78(1.13, 6.84)	0.162
TNF **rs1800629**															
AA/AG	469	0.98(0.28, 3.48)	0.974	464	1.06(0.46, 2.48)	0.889	466	0.72(0.22, 2.3)	0.692	465	0.93(0.54, 1.61)	0.931	461	0.81(0.17, 3.87)	0.792
GG	875	1.92(0.83, 4.47)	0.360	856	1.24(0.74, 2.06)	0.830	869	1.78(0.90, 3.53)	0.264	863	0.98(0.65, 1.48)	0.931	850	1.91(0.89, 4.09)	0.190

[a]Adjusted for study, city, intervention, gender, maternal age at birth, maternal smoking during pregnancy, environmental tobacco smoke in the home, birth weight and parental atopy. [b]P-values were corrected for multiple testing using the Bonferroni method.

Supplemental Material, Table S9. Main genetic and environmental effects for asthma and wheeze at school age; and association between traffic-related NO_2 and asthma and wheeze at school age, stratified by genotype (CAPPS – Vancouver only, n = 173).

Model	Current asthma			Ever asthma			Current wheeze			Ever wheeze			Ever asthma and current wheeze		
	N	aOR[a] (95%CI)	p-val[b]	N	aOR[a] (95%CI)	p-val[b]	N	aOR[a] (95%CI)	p-val[b]	N	aOR[a] (95%CI)	p-val[b]	N	aOR[a] (95%CI)	p-val[b]
Main Effects															
GSTP1 rs1138272 TT/TC v. CC	173	0.73(0.16, 3.36)	0.688	173	1.24(0.49, 3.11)	0.795	173	0.84(0.26, 2.71)	0.767	173	0.69(0.26, 1.85)	0.698	173	0.85(0.23, 3.13)	0.811
GSTP1 rs1695 GG/GA v. AA	171	0.43(0.14, 1.29)	0.347	171	0.91(0.47, 1.8)	0.795	171	0.41(0.16, 1.02)	0.165	171	0.97(0.49, 1.93)	0.94	171	0.32(0.11, 0.97)	0.129
TNF rs1800629 AA/AG v. GG	172	1.99(0.65, 6.15)	0.347	172	1.55(0.75, 3.21)	0.714	172	1.62(0.64, 4.09)	0.462	172	1.49(0.70, 3.16)	0.698	172	2.02(0.73, 5.59)	0.269
Traffic-related NO_2 (per $10\mu g/m^3$)	173	1.93(0.92, 4.03)	0.08	173	1.60(0.93, 2.77)	0.092	173	1.31(0.70, 2.46)	0.402	173	1.26(0.74, 2.16)	0.398	173	1.77(0.93, 3.36)	0.08
Stratified Results															
***GSTP1* rs1138272**															
TT/TC	25	2.30(0.02, 256)	0.728	25	1.46(0.24, 8.71)	0.814	25	0.08(0.01, 6.82)	0.528	25	0.97(0.17, 5.53)	0.975	25	6.18(0.03, 1293)	0.504
CC	148	2.32(0.99, 5.42)	0.195	148	1.96(1.01, 3.78)	0.276	148	1.82(0.90, 3.72)	0.327	148	1.40(0.73, 2.7)	0.878	148	2.60(1.26, 5.38)	0.060
***GSTP1* rs1695**															
GG/GA	81	4.05(0.92, 17.9)	0.195	81	1.71(0.81, 3.62)	0.324	81	0.84(0.35, 2.02)	0.703	81	0.88(0.42, 1.84)	0.938	81	1.83(0.79, 4.27)	0.264
AA	90	1.44(0.40, 5.12)	0.691	90	1.80(0.64, 5.11)	0.401	90	2.62(0.81, 8.47)	0.327	90	2.53(0.85, 7.51)	0.570	90	2.29(0.69, 7.61)	0.264
***TNF* rs1800629**															
AA/AG	43	4.02(0.45, 35.7)	0.424	43	1.13(0.24, 5.3)	0.877	43	1.94(0.34, 11.1)	0.687	43	0.81(0.18, 3.63)	0.938	43	4.13(0.58, 29.3)	0.264
GG	129	1.34(0.53, 3.42)	0.691	129	1.67(0.87, 3.21)	0.324	129	1.21(0.55, 2.64)	0.703	129	1.32(0.65, 2.66)	0.878	129	1.47(0.69, 3.13)	0.383

[a]Adjusted for study, city, intervention, gender, maternal age at birth, maternal smoking during pregnancy, environmental tobacco smoke in the home, birth weight and parental atopy. [b]P-values were corrected for multiple testing using the Bonferroni method.

Supplemental Material, Table S10. Main genetic and environmental effects for asthma and wheeze at school age; and association between traffic-related NO_2 and asthma and wheeze at school age, stratified by genotype (CAPPS & SAGE – Winnipeg only, n = 351).

Model	Current asthma			Ever asthma			Current wheeze			Ever wheeze			Ever asthma and current wheeze		
	N	aOR[a] (95%CI)	p-val[b]	N	aOR[a] (95%CI)	p-val[b]	N	aOR[a] (95%CI)	p-val[b]	N	aOR[a] (95%CI)	p-val[b]	N	aOR[a] (95%CI)	p-val[b]
Main Effects															
GSTP1 rs1138272 TT/TC v. CC	347	2.21(1.14, 4.27)	0.057	347	2.08(1.12, 3.89)	0.063	286	0.59(0.22, 1.58)	0.856	286	0.46(0.19, 1.1)	0.237	286	1.99(0.95, 4.2)	0.210
GSTP1 rs1695 GG/GA v. AA	347	1.35(0.82, 2.23)	0.360	347	1.06(0.67, 1.67)	0.958	286	0.93(0.43, 2.02)	0.856	286	0.83(0.40, 1.72)	0.865	286	1.26(0.71, 2.24)	0.642
TNF rs1800629 AA/AG v. GG	351	1.18(0.70, 2)	0.542	351	1.01(0.62, 1.65)	0.958	289	0.92(0.39, 2.18)	0.856	289	0.93(0.41, 2.12)	0.865	289	1.01(0.56, 1.84)	0.97
Traffic-related NO_2 (per $10\mu g/m^3$)	351	0.91(0.45, 1.83)	0.784	351	1.32(0.71, 2.46)	0.377	289	1.34(0.49, 3.65)	0.562	289	0.87(0.34, 2.21)	0.766	289	0.87(0.39, 1.93)	0.735
Stratified Results															
***GSTP1* rs1138272**															
TT/TC	52	19.2(0.79, 466)	0.138	52	3.55(0.22, 57.5)	0.776	43	158(3.04, 8222)	0.072	43	54.5(1.20, 2480)	0.177	43	4.21(0.25, 69.9)	0.534
CC	295	0.48(0.22, 1.03)	0.138	295	1.01(0.51, 1.98)	0.986	243	0.73(0.24, 2.22)	0.618	243	0.53(0.19, 1.51)	0.351	243	0.55(0.22, 1.37)	0.534
***GSTP1* rs1695**															
GG/GA	190	1.81(0.71, 4.65)	0.324	190	1.82(0.72, 4.64)	0.776	159	2.39(0.59, 9.71)	0.437	159	1.43(0.40, 5.08)	0.692	159	1.57(0.54, 4.58)	0.534
AA	157	0.27(0.08, 0.9)	0.138	157	0.96(0.36, 2.53)	0.986	127	0.37(0.07, 1.82)	0.437	127	0.24(0.05, 1.06)	0.177	127	0.40(0.09, 1.75)	0.534
***TNF* rs1800629**															
AA/AG	93	1.17(0.26, 5.22)	0.839	93	1.23(0.32, 4.79)	0.986	80	0.29(0.03, 2.9)	0.437	80	0.22(0.03, 1.67)	0.288	80	0.51(0.09, 2.85)	0.534
GG	258	0.83(0.35, 1.96)	0.803	258	1.39(0.66, 2.94)	0.776	209	1.37(0.40, 4.68)	0.618	209	0.83(0.28, 2.5)	0.741	209	0.97(0.35, 2.7)	0.949

[a]Adjusted for study, city, intervention, gender, maternal age at birth, maternal smoking during pregnancy, environmental tobacco smoke in the home, birth weight and parental atopy. [b]P-values were corrected for multiple testing using the Bonferroni method.

Supplemental Material, Table S11. Main genetic effects of *GSTP1* and *TNF* for asthma and wheeze at school age, for children not in an intervention arm (pooled data, n = 3,695).

Genotype	Current asthma			Ever asthma			Current wheeze			Ever wheeze			Ever asthma and current wheeze		
	N	aOR[a] (95%CI)	p-val[b]	N	aOR[a] (95%CI)	p-val[b]	N	aOR[a] (95%CI)	p-val[b]	N	aOR[a] (95%CI)	p-val[b]	N	aOR[a] (95%CI)	p-val[b]
GSTP1 rs1138272 TT/TC v. CC	3641	1.63(1.15, 2.32)	0.021	3549	1.48(1.16, 1.89)	0.006	3537	1.03(0.71, 1.48)	0.881	3552	0.97(0.79, 1.21)	0.807	3468	1.46(1.06, 2.02)	0.060
GSTP1 rs1695 GG/GA v. AA	3695	0.92(0.69, 1.22)	0.592	3630	0.93(0.77, 1.14)	0.640	3614	0.98(0.74, 1.28)	0.881	3606	0.96(0.81, 1.13)	0.807	3549	0.95(0.73, 1.23)	0.681
TNF rs1800629 AA/AG v. GG	3572	1.09(0.80, 1.49)	0.592	3484	1.05(0.85, 1.30)	0.640	3469	1.20(0.90, 1.61)	0.672	3482	1.19(1.00, 1.41)	0.150	3402	1.15(0.86, 1.53)	0.514

[a]Adjusted for study, city, gender, maternal age at birth, maternal smoking during pregnancy, environmental tobacco smoke in the home, birth weight and parental atopy. [b]P-values were corrected for multiple testing using the Bonferroni method.

Supplemental Material, Table S12. Main genetic and environmental effects for asthma and wheeze at school age; and association between traffic-related NO_2 and asthma and wheeze at school age, stratified by genotype (pooled data excluding non-Caucasian children in CAPPS and SAGE, n = 4,821).

**Model**	**Current asthma**			**Ever asthma**			**Current wheeze**			**Ever wheeze**			**Ever asthma and current wheeze**		
	**N**	**aOR[a] (95%CI)**	**p-val[b]**	**N**	**aOR[a] (95%CI)**	**p-val[b]**	**N**	**aOR[a] (95%CI)**	**p-val[b]**	**N**	**aOR[a] (95%CI)**	**p-val[b]**	**N**	**aOR[a] (95%CI)**	**p-val[b]**
Main Effects															
GSTP1 rs1138272 TT/TC v. CC	4309	1.63(1.18, 2.25)	0.009	4178	1.50(1.20, 1.87)	0.000	4203	1.20(0.89, 1.62)	0.363	4206	1.04(0.87, 1.25)	0.661	4097	1.63(1.21, 2.19)	0.003
GSTP1 rs1695 GG/GA v. AA	4457	0.91(0.69, 1.18)	0.46	4344	0.92(0.77, 1.1)	0.551	4375	1.00(0.79, 1.27)	0.992	4348	0.94(0.82, 1.08)	0.567	4263	0.95(0.74, 1.23)	0.705
TNF rs1800629 AA/AG v. GG	4203	1.27(0.96, 1.68)	0.138	4076	1.05(0.87, 1.27)	0.622	4098	1.23(0.96, 1.58)	0.327	4099	1.16(1.00, 1.34)	0.171	3994	1.15(0.88, 1.51)	0.440
Traffic-related NO_2 (per 10µg/m^3)	4821	1.26(0.96, 1.65)	0.093	4671	1.27(1.06, 1.52)	0.008	4712	1.08(0.86, 1.37)	0.512	4707	1.02(0.89, 1.16)	0.811	4587	1.14(0.86, 1.53)	0.367
Stratified Results															
***GSTP1* rs1138272**															
TT/TC	757	2.76(1.50, 5.07)	0.006	737	1.63(1.05, 2.52)	0.074	740	1.08(0.61, 1.93)	0.816	743	1.05(0.74, 1.48)	0.796	725	1.41(0.70, 2.82)	0.636
CC	3552	1.04(0.74, 1.47)	0.805	3441	1.22(0.99, 1.51)	0.094	3463	1.06(0.78, 1.42)	0.816	3463	1.07(0.91, 1.27)	0.606	3372	1.16(0.81, 1.66)	0.636
***GSTP1* rs1695**															
GG/GA	2590	1.52(1.10, 2.12)	0.036	2527	1.38(1.09, 1.74)	0.042	2549	1.19(0.87, 1.61)	0.816	2536	1.11(0.93, 1.33)	0.606	2486	1.20(0.82, 1.74)	0.636
AA	1867	0.93(0.57, 1.52)	0.805	1817	1.18(0.87, 1.6)	0.291	1826	0.91(0.61, 1.37)	0.816	1812	0.94(0.74, 1.19)	0.720	1777	1.04(0.62, 1.75)	0.882
***TNF* rs1800629**															
AA/AG	1346	1.48(0.94, 2.33)	0.174	1307	1.44(1.02, 2.03)	0.074	1321	0.95(0.60, 1.5)	0.816	1321	1.13(0.86, 1.49)	0.606	1288	1.17(0.71, 1.95)	0.640
GG	2857	1.16(0.77, 1.73)	0.714	2769	1.22(0.97, 1.55)	0.112	2777	1.25(0.91, 1.71)	0.816	2778	1.08(0.91, 1.3)	0.606	2706	1.18(0.79, 1.74)	0.636

[a]For a 10 µg/m^3 increase in NO_2. Adjusted for study, city, intervention, gender, maternal age at birth, maternal smoking during pregnancy, environmental tobacco smoke in the home, birth weight and parental atopy. [b]P-values were corrected for multiple testing using the Bonferroni method.
